# Comparative Proteomic Studies of *Yersinia pestis* Strains Isolated from Natural Foci in the Republic of Georgia

**DOI:** 10.3389/fpubh.2015.00239

**Published:** 2015-10-16

**Authors:** Maia Nozadze, Ekaterine Zhgenti, Maia Meparishvili, Lia Tsverava, Tamar Kiguradze, Gvantsa Chanturia, Giorgi Babuadze, Merab Kekelidze, Lela Bakanidze, Tatiana Shutkova, Paata Imnadze, Stephen C. Francesconi, Richard Obiso, Revaz Solomonia

**Affiliations:** ^1^Institute of Chemical Biology, Ilia State University, Tbilisi, Georgia; ^2^I.Beritashvili Center for Experimental Biomedicine, Tbilisi, Georgia; ^3^National Center for Disease Control, Tbilisi, Georgia; ^4^Naval Medical Research Center, Frederick, MD, USA; ^5^Attimo Research and Development, Blacksburg, VA, USA

**Keywords:** *Yersinia pestis*, proteome, 2-D gel electrophoresis, virulence, Republic of Georgia

## Abstract

*Yersinia pestis*, the causative agent of plague, is a highly virulent bacterium responsible for millions of human deaths throughout history. In the last decade, two natural plague foci have been described in the Republic of Georgia from which dozens of *Y. pestis* strains have been isolated. Analyses indicate that there are genetic differences between these strains, but it is not known if these differences are also reflected in protein expression. We chose four strains of *Y. pestis* (1390, 1853, 2944, and 8787) from the National Center for Disease Control and Public Health collection for proteomic studies based on neighbor-joining tree genetic analysis and geographical loci of strain origin. Proteomic expression was analyzed using two-dimensional gel electrophoresis and mass spectrometry. Select *Y. pestis* strains were grown under different physiological conditions and their proteomes were compared: (1) 28°C without calcium; (2) 28°C with calcium; (3) 37°C without calcium; and (4) 37°C with calcium. Candidate proteins were identified and the differences in expression of F1 antigen, tellurium-resistance protein, and outer membrane protein C, porin were validated by Western blotting. The *in vitro* cytotoxicity activity of these strains was also compared. The results indicate that protein expression and cytotoxic activities differ significantly among the studied strains; these differences could contribute to variations in essential physiological functions in these strains.

## Introduction

*Yersinia pestis* is a Gram-negative, rod-shaped bacterium that is the causative agent of plague, a high-mortality disease recorded throughout history. Most human *Y. pestis* infections are flea-borne and manifest as bubonic plague; the other two forms of plague, septicemic and pneumonic, are rare. Public health experts recognize plague as a re-emerging infectious disease. In addition, *Y. pestis* carries the potential for use as a biological weapon because it can be mass-produced, is easily aerosolized, and may result in highly fatal pneumonic plague, which can spread person-to-person via inhalation of infectious air-borne droplets (1, 2).

Strains of *Y. pestis* isolated from different natural foci exhibit distinct biochemical and virulent phenotypes (1); laboratory passaging is also known to influence the accumulation of genetic polymorphisms in plague vaccine strains (3). In the event of infectious disease outbreaks, detailed knowledge of the molecular characteristics of *Y. pestis* strains is important to enable epidemiological traceability.

Several factors are thought to contribute to the persistence of *Y. pestis* in the environment. The stability of the rodent-flea infection cycle allows for constant circulation of the organism within susceptible host species (4). In this repeating cycle of rodent-flea infection, the organism is capable of causing fatal disease in Muridae and Sciuridae populations, and fleas must continually infect new hosts to find new blood meals (5). *Y. pestis* is therefore dependent on vectors and reservoirs for continued enzootic replication.

The longevity of fleas infected with *Y. pestis* also contributes to the duration of the enzootic cycle of infection; these insect reservoirs can sustain enzootic foci over long periods before their death, even when the host rodents are asymptomatic (6). In addition, the longevity of certain species of rodents post-infection can play a role in enzootic cycles of infection: certain rodent species, including some within the *Microtus* (7–10) and *Meriones* (11–14) genera, exhibit moderate- to high-resistance to plague and can carry *Y. pestis* without quickly dying (15). Some isolates of *Y. pestis* are less virulent than others, and this decreased virulence is thought to contribute to the stable enzootic circulation of *Y. pestis* because host and reservoir live longer and therefore have more opportunities to transmit the organism (16). In ancient plague foci, such as those in the Caucasus, vector–host cycles with different transmission and disease dynamics may co-exist (17) in which *Y. pestis* variants are highly virulent in certain rodents but avirulent in man (16).

Plague re-emergence in areas where the disease has been absent for many decades is not well understood (18–20). Two different mechanisms have been proposed: either *Y. pestis* continues circulating in animal populations at undetectable levels, or it is reintroduced to the animal population (21, 22). Although *Y. pestis* evolved from the enteric bacterium *Yersinia pseudotuberculosis*, which can survive for long periods in soil and water, selective pressure exerted by vector-borne transmission has resulted in the loss of such functionality (18, 23, 24). *Y. pestis* lacks the survival traits of other yersiniae, and long-term persistence outside of a host or vector is unlikely.

Plague epidemics have been recorded throughout the last century in the Republic of Georgia. During the last 50 years, two natural foci have been described from which multiple *Y. pestis* strains have been isolated (3). These foci are located in the Transcaucasian highland (encompassing the Ninotsminda and Akhalkalaki regions on the Javakheti plateau) and the Iori region (encompassing eastern Georgia and the border between Georgia and Azerbaijan) (25).

*Yersinia pestis* was isolated for the first time in Georgia in the spring of 1966, coinciding with a rodent epizootic of plague in several foci within the country. The cultures were isolated from the carcasses of *Meriones libycus* (red gerbils) and their fleas, *Xenopsylla conformis* and *Ceratophyllus laeviceps*. Strains were isolated from Eldari, Nazarlebi, and Taribani steppes, and the existence of a natural focus of plague among a Georgian population of *M. libycus* was confirmed for the first time. Later studies showed that *M. libycus* is the plague reservoir in this focus and the primary vectors are *X. conformis* and *C. laeviceps* fleas (26, 27).

The Transcaucasian desert plague foci are spread across approximately 500,000 ha, with each focus covering 40,000–200,000 ha. The foci are in a semi-desert zone near the Iori and Alazani rivers. Epizootics are severe in this area and *Y. pestis* strains that are isolated tend to be highly virulent; most of these strains were isolated from dead rodents. The epizootic process is spread across several individual foci with the same geographic features.

Georgian Anti-Plague Stations have conducted active surveillance in these foci since the early 1900s and have collected over 120 *Y. pestis* strains from these regions (28). The National Center for Disease Control and Public Health (NCDCPH) collection currently contains 46 strains, 40 of which were isolated from Georgian plague foci between 1966 and 1997 (25).

The differences between *Y. pestis* strains, including Georgian strains, on a genomic level have been extensively studied (25, 29–32). It is likely that some of these strain variations are also reflected at the proteomic level as the final products of gene expression. At the time of publication, no comparative studies of trans-Caucasian *Y. pestis* strains at the proteomic level were available in published literature.

A neighbor-joining tree was generated using multilocus variable number tandem repeat analysis for the 46 unique *Y. pestis* strains in the NCDCPH collection in Tbilisi, Georgia (30). Four strains of *Y. pestis* located at different positions on this tree were selected for comparative proteomic analysis.

Early studies of *Yersinia* physiology uncovered the low calcium response, whereby bacterial cultures grown in rich medium at an elevated temperature (37°C) exhibit a growth defect upon chelation of calcium ions. This defect was shown to be a result of a type III secretion system in *Y. pestis*, the Ysc TTSS, and is responsible for the secretion of virulence factors known as *Yersinia* outer proteins (YOPS) (33). This secretion system can be activated *in vitro* and virulence factors can be released into the medium when *Y. pestis* is grown at 37°C in the presence of millimolar concentrations of calcium (34). The *Y. pestis* proteome was previously examined using two-dimensional (2-D) gel electrophoresis (35, 36). These studies showed that virulence factors were induced at 26 or 37°C in the presence of 2.5 mM Ca^2+^ (a concentration similar to that in mammalian plasma). The *Y. pestis* proteome varies as a function of temperature and calcium, and expression of virulence factors clearly depends on these physiological conditions (37); thus, differences between the proteomes of *Y. pestis* strains could be masked under one set of physiological conditions, but expressed under another.

In this study, we examined and characterized proteomes of *Y. pestis* strains from the trans-Caucasian area as a function of temperature and calcium, which were used to affect induction of virulence. *Y. pestis* strains were grown at different temperatures in the presence and absence of calcium ions (Ca^2+^) and their proteomes were compared by 2-D gel electrophoresis and mass spectrometry (MS) and confirmed by Western blotting.

## Materials and Methods

### *Yersinia pestis* Strains

The following strains of *Y. pestis* were chosen for comparative proteomic studies: 1390, 1853, 8787, and 2944. These strains were provided by the NCDCPH in Tbilisi, Georgia. Strains 1390, 1853, and 8787 were isolated in Ninotsminda, Georgia, in 1979, 1980, and 1992, respectively; strain 2944 was isolated in Kabardino-Balkaria, Russia, in 1975.

### *Yersinia pestis* Growth with Temperature and Calcium Concentration Changes

The following procedure was carried out in quadruplicate for each *Y. pestis* strain included in the study. Cultures were grown overnight in Mueller-Hilton broth at 28°C with continuous shaking. After incubation, 0.1 mL of each culture was transferred into 15 mL of fresh broth. Two aliquots were incubated at 37°C, one of which was adjusted with 0.4 M CaCl_2_ (15 μL) to a final concentration of 4 mM; 15 μL of sterile distilled water was added to the second aliquot. The remaining two aliquots were treated as described above but incubated at 28°C. All aliquots were incubated for another 4 h at the specified temperatures. Cells were harvested during the exponential phase of growth. Aliquots were centrifuged at 3,000 × *g* for 10 min, and duplicate bacterial pellets from strains under each set of physiological conditions were prepared for one-dimensional (1-D) sodium dodecyl sulfate polyacrylamide gel electrophoresis (SDS-PAGE) and 2-D gel electrophoresis.

#### Preparation for 1-D SDS-PAGE

Bacterial pellets were resuspended in 5% SDS and incubated at 95°C for 10 min. Lysates were centrifuged at 15,000 × *g* for 15 min, and the supernatant was collected and tested for sterility; inactivated samples were inoculated and grown in tryptic soy broth with 0.0125% phenol red for 72 h at 28°C. After incubation, samples were assessed for color change or turbidity; samples were considered sterile if neither color change nor turbidity were observed.

#### Preparation for 2-D Gel Electrophoresis

Bacterial pellets were resuspended in buffer (25 mM Tris-acetate, pH 7.8, 5 mM EDTA, 150 μg/mL lysozyme, 2 mM PMSF, 0.05% Triton X-100, and 1 mM benzamidine). Samples were incubated for 30 min at 20°C with intermittent vortexing. Lysates were centrifuged at 20,000 × *g* for 60 min at 4°C. The supernatant was collected and 20% CHAPS stock solution was added to a final concentration of 2% (w/v), followed by incubation at 95°C for 10 min. After incubation, the supernatant was tested for sterility as previously described.

### Protein Quantification

A micro-BCA kit (Pierce, Thermo Scientific) was used to quantify the protein concentration in supernatants. Protein quantification was performed in quadruplicate for each sample with the appropriate buffer controls, according to the manufacturer’s instructions.

### 2-D Gel Electrophoresis

#### Sample Preparation and Isoelectrofocusing

Isoelectrofocusing (IEF) strips (linear pH 3–10) were rehydrated in 8M urea, 0.5% Triton X-100, 0.5% Pharmalyte 3–10, and 30 mM DTT overnight. Protein samples (30 μg) were loaded onto rehydrated strips in buffer containing 7M urea, 2M thiourea, 2% CHAPS, 2% Triton X-100, 0.1% ASB-14, 2*-*mercaptoethanol, 2% Pharmalyte 3–10, and bromophenol blue. IEF was carried out at 500 V for 3 h and 3,500 V for 18 h ± 30 min.

#### Equilibration

Strips were equilibrated for 15 min in a buffer containing 0.05M Tris-HCl (pH 6.8), 6M urea, 30% glycerol, 3% SDS, and 1% DTT, followed by equilibration in the same buffer with 2.5% DTT iodoacetamide instead of 1% DTT for 15 min.

#### SDS Electrophoresis

SDS electrophoresis was run on a 1-mm thick, 12.5% polyacrylamide gel at 25°C, first at 10 mA per gel at 80 V for 1 h, and then at 12 mA per gel at 150 V for 17 h.

#### Staining, Scanning, and Analysis

The gels were stained with a silver stain kit (GE-Healthcare), omitting the glutaraldehyde step. Silver-stained gels were scanned with an image scanner (Labscan 6.0). Images were digitalized and processed using Image Master 2D platinum 7.0.

In each series of experiments, the supernatants of the four *Y. pestis* strains were analyzed concurrently for each physiological condition. Proteins that exhibited at least 1.5-fold difference between the strains were selected. The relative intensities of protein spots coinciding by location (isoelectric point and molecular weight) from different experiments were compared by *t*-test. The significantly differentially expressed protein spots (*p* < 0.05) were excised, destained, and stored at −20°C until MS analysis.

### In-Gel Digestion and MS Analysis

Excised proteins were reduced with TCEP and alkylated with iodoacetamide. Samples were then treated with acetonitrile, dried, and rehydrated in activated trypsin (Thermo Scientific, Pierce) to begin digestion; proteins were digested at 37°C for 4 h. Digested samples were processed using nanospray ionization tandem HPLC-MS/MS CID performed with helium (Finnigan LTQ, Thermo Scientific). MS/MS spectra data was analyzed using SEQUEST (Proteome Discoverer 2.0), searching against UniProt UniRef100 databases.

### Antibodies

Antibodies against F1 antigen were obtained from Life Science-Meridian (cat. # C86308M). Polyclonal antibodies against DNA-binding protein H-NS, tellurium-resistance protein, and outer membrane protein C, porin, were raised in rabbits against the following peptide sequences:

- H-NS: EMLEKLEVVVN (amino acids 28–38)
- Tellurium-resistance protein: PADVDKIVFVVT (amino acids 99–110)
- Outer membrane protein C, porin: NTDDIVAVGMVYQ (amino acids 332–344)

Antibodies against H-NS and tellurium-resistance protein were affinity-purified on corresponding peptide columns; antibodies against outer membrane protein C, porin were purified on protein-A Sepharose columns. The specificities of antibodies were tested by inhibition of binding with immunizing peptides using Western blotting.

### 1-D SDS-PAGE and Western Blotting

Laemli SDS sample buffer was added to protein aliquots (30 μg). Samples were then analyzed by SDS-PAGE, followed by Western blotting. After electrophoretic transfer onto nitrocellulose membranes, protein bands were detected by staining with Ponceau S to confirm the uniform loading of the gel. Nitrocellulose membranes were sectioned according to the molecular weight standard and were then probed with primary antibodies against the following antigens: F1; tellurium-resistance protein; outer membrane protein C, porin; and DNA-binding protein H-NS. Because F1 and outer membrane protein C, porins are of a similar molecular weight, separate electrophoresis were carried out for each antigen.

Standard immunochemical procedures were carried out, and bands were visualized using peroxidase-labeled secondary antibodies (anti-rabbit) and a SuperSignal West Pico Chemiluminescent Substrate (Thermo Scientific, Pierce). Intensifying screens were used to expose the blots to X-ray films pre-flashed with Sensitize (Amersham). The densities of the protein bands were measured using LabWorks 4.0 software (UVP).

The autoradiographs were calibrated using combined protein from the four strains (15, 30, 45, and 60 μg total protein). Standard curves were prepared for each protein by plotting the optical density of the immunostained band against the protein concentration. The least squares regression showed a significant fit to a straight line for all standards. In experimental samples, to calculate protein amount, the optical density of each band was divided by the optical density of the 30-μg total protein standard; this value was considered to be the “relative amount” of protein (e.g., F1, Tellurium-resistance protein, and H-NS) (38).

### *In vitro* Cytotoxicity Assay

*In vitro* cytotoxicity of *Y. pestis* strains and the vaccine strain EV was evaluated based on strain ability to induce apoptosis in macrophage cultures. Apoptosis was assayed using a colorimetric caspase-3 assay kit (Sigma-Aldrich, cat. #CASP-3-C) according to the manufacturer’s instructions. This kit measures the activity of caspase-3, one of the critical enzymes of apoptosis, and includes a specific inhibitor for precise measurement of caspase-3 activity.

Macrophages were cultivated as previously described (39, 40). Briefly, J774A.1 mouse macrophages were grown in DMEM supplemented with 10% heat-inactivated FBS. Cultures were incubated for 72 h at 37°C in a 5% CO_2_ atmosphere, after which the wells were washed to remove non-adherent cells.

To each well, 100 μL of *Y. pestis* (3 × 10^8^ cfu/mL) was added and incubated at 37°C for 3 h. Plates were centrifuged at 600 × *g* for 5 min at 4°C, washed once with 1 mL of PBS per well, and 1× lysis buffer was added (100 μL/10^5^cells). After 15 min of incubation on ice, the lysate was centrifuged at 3,000 × *g* for 5 minutes at 4°C, and the supernatant was stored at −70°C. Supernatants were filtered, divided into aliquots, and tested for sterility as previously described. Cell lysates and positive controls were brought to the required volume with 1× assay buffer and incubated for 2 h at 37°C with caspase-3 substrate. For each aliquot, caspase-3 inhibitor was included in addition to the peptide substrate, and parallel measurements were taken. The amount of *p*-nitroaniline (pNA) released in the assay was measured using a spectrophotometer (OD_405 nm_) and the concentration was determined by the standard curve. These values were subtracted from the values obtained without the inhibitor. The protein amount was determined in cell lysates, and enzyme activity expressed as nanomoles of pNA released per minute per 1 mg of cell lysate protein.

### Statistical Analysis

The relative amounts of proteins were subjected to analysis of variance (ANOVA) by strain (1390, 1853, 2944, and 8787) and physiological condition (28°C, 28°C with Ca^2+^, 37°C, 37°C with Ca^2+^). Results from different strains grown under the same physiological conditions were compared by *t*-test.

Caspase-3 activity data was analyzed by strain using one-way ANOVA. Planned comparisons were done by *t*-test. All *t*-tests were two-tailed unless otherwise indicated.

## Results

### 2-D Gel Electrophoresis

**Figure 1:**
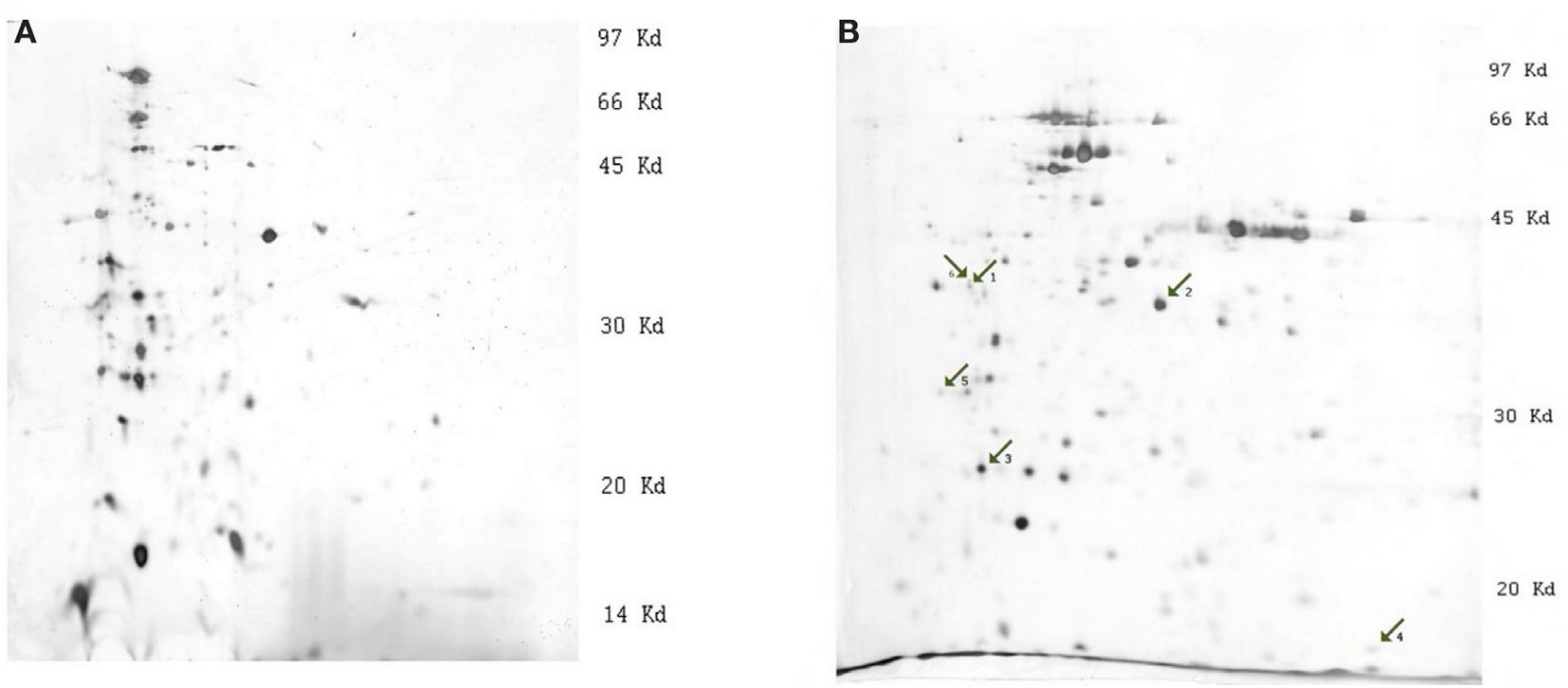
**Images of silver stained 2-D gels of *Y. pestis* protein extracts (strain 1853, condition 28°C)**. **(A)** 2-D electrophoresis on strips with pH linear gradient 3.0–10.0, **(B)** 2-D electrophoresis on strips with pH linear gradient 3.0–5.6. The numbered arrows indicate the positions of differentially expressed proteins: 1, outer membrane protein C, porin; 2, outer membrane protein C2 porin; 3, Tellurium-resistance protein; 4, DNA-binding protein H-NS; and 5, F1 capsule antigen.

The 2-D gel electrophoresis of *Y. pestis* protein extracts was carried out on IEF strips with two pH gradients: 3.0–10.0 and 3.0–5.6 (Figures 1A,B). The majority of the proteins on the 3.0–10.0 pH gradient gels were concentrated between pH 3.0 and 6.0 (Figure 1A), so 2-D gel electrophoresis was repeated using strips with a pH gradient from 3.0 to 5.6 for better resolution of protein bands (Figure 1B).

The comparison of images of different strains revealed consistent differential expression of bands. The most significant differences were observed at 28°C. Strains were divided into two groups according to their differences: (1) 1853 and 1390 and (2) 2944 and 8787. Differential expression of certain proteins was significantly higher in the first group of bacterial strains. MS analysis was used to determine the identity of the excised proteins (Table 1). To validate the significant differences between the proteins, quantitative Western blotting was used to study four of the five identified proteins. The criteria for the selection of proteins that we studied were the following reasons: (i) availability of the commercial antibodies, (ii) our ability to produce custom antibodies, and (iii) differential expression profiles of these proteins under the various growth conditions.

**Table 1:** **Differentially expressed proteins identified by 2-D electrophoresis by condition and strain**.

Protein	Condition/strain
Outer membrane protein C, porin	28°C, 1853 and 1390 > 2944, 8787
Tellurium-resistance protein	28°C, 1853 and 1390 > 2944, 8787
DNA-binding protein H-NS	28°C, 1853 and 1390 > 2944, 8787
F1 antigen	37°C, 37°C + Ca^2+^ and 28°C, 1853 and 1390 > 2944, 8787
Outer membrane protein-C2, porin	28°C, 1853 and 1390 > 2944, 8787

### Immunostaining

**Figure 2:**
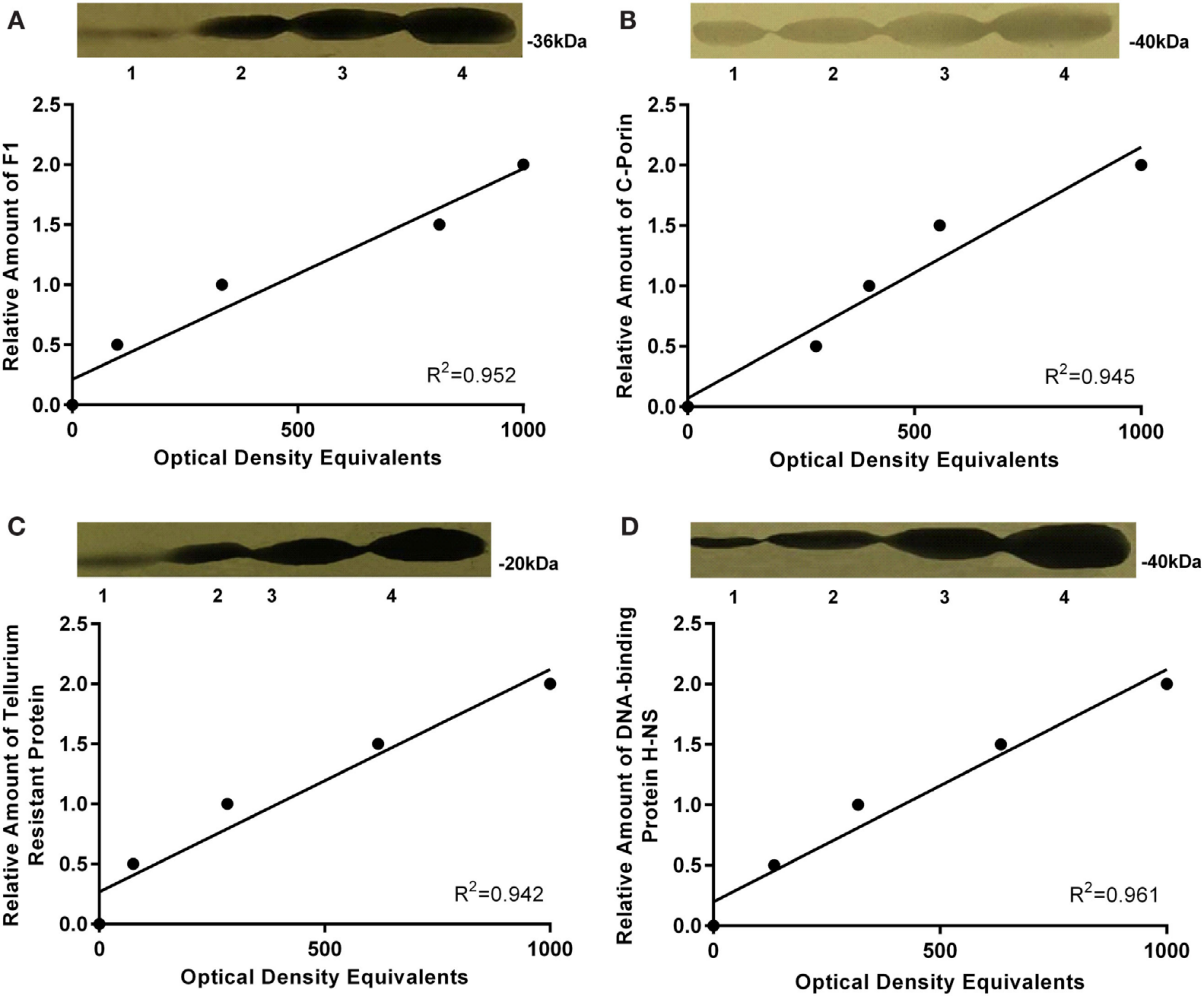
**Western blot autoradiographs and calibration plots**. Western blot autoradiographs for single standard sample of the mixture of all *Y. pestis* strains protein extracts containing 15, 30, 45, and 60 μg of protein, respectively. Calibration plots are shown below each autoradiograph and reflect the linear response of immunostaining with the corresponding amount of loaded protein for **(A)** F1 antigen, **(B)** outer membrane protein C, porin, **(C)** Tellurium, and **(D)** DNA-binding protein H-NS.

Standards were prepared as described above (15, 30, 45, and 60 μg total protein). For these standards, the optical densities of the bands immunostained for F1, outer membrane protein C, porin, tellurium-resistance protein, and DNA-binding protein H-NS were linearly related to the amounts of proteins in the bands (Figures 2A–D).

Antibodies against F1 reacted with a protein band of molecular weight of approximately 36–38 kDa (Figure 2A). The molecular weight of F1 antigen is 17.7 kDa, but it migrates as a dimer in SDS electrophoresis (41). Outer membrane protein C, porin antibodies reacted with a protein band of apparent molecular weight approximately 38 kDa, which corresponds to the expected size of the target protein (Figure 2B). Tellurium-resistance protein antibodies reacted with a protein band of 20 kDa (Figure 2C) and DNA-binding protein H-NS antibodies reacted with a protein of apparent molecular weight 15–16 kDa (Figure 2D). Both of these weights corresponded to the expected size of the target proteins.

### F1 Antigen

**Figure 3:**
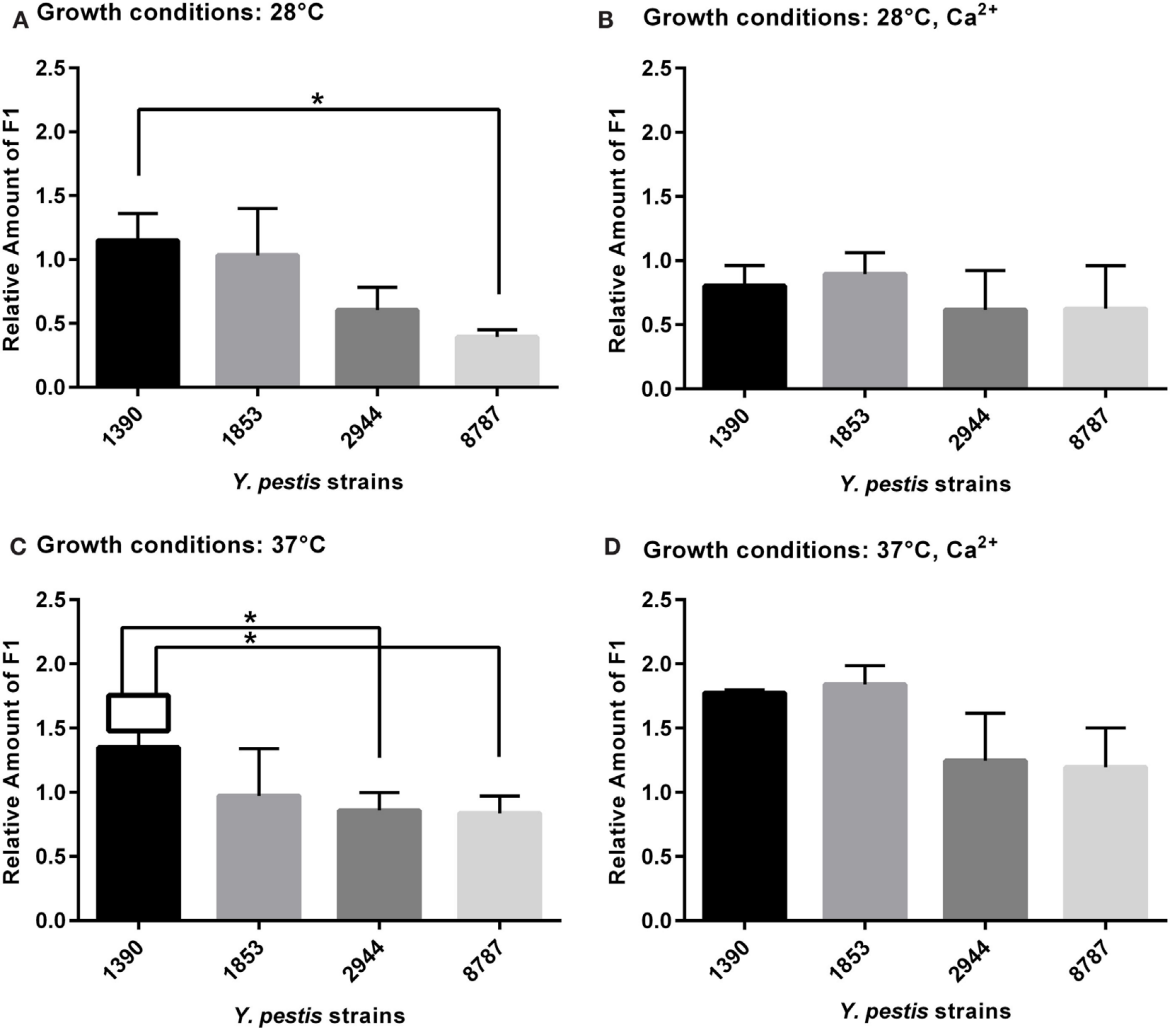
**Relative amount of F1 antigen expressed by *Y. pestis* strains 1390, 1853, 2944, and 8787 at physiological growth conditions (A) 28°C; (B) 28°C, Ca^2+^; (C) 37°C; and (D) 37°C, Ca^2+^**. The relative amount of protein expressed was calculated from immunostained bands, by dividing the optical density of the band by the optical density of the corresponding 30 μg total protein standard. Data represents the mean + SEM. *Indicates significant differences (*p* < 0.05).

*Y. pestis* strain and growth condition were both found to be significant factors in the expression of F1 when analyzed by two-way ANOVA (respectively *F*_3, 63_ = 5.40, *p* = 0.002; *F*_3, 63_ = 9.95, *p* = 0.0001). As expected from 2-D electrophoresis data, the most significant changes were observed at 37°C (Figure 3C). The mean amount of F1 antigen was significantly higher in strain 1390 than in strains 2944 (*t* = 2.65, *p* = 0.038) and 8787 (*t* = 2.85, *p* = 0.029). The mean amount of F1 was also higher in strain 1390 than strain 8787 at 28°C (*t* = 3.47, *p* = 0.013; Figure 3A). At 28°C with Ca^2+^, and 37°C with Ca^2+^, mean amounts of F1 antigen in 1390 and 1853 were not significantly higher than mean amounts in 8787 and 2944 (Figures 3B,D, respectively).

### Outer Membrane Protein C, Porin

**Figure 4:**
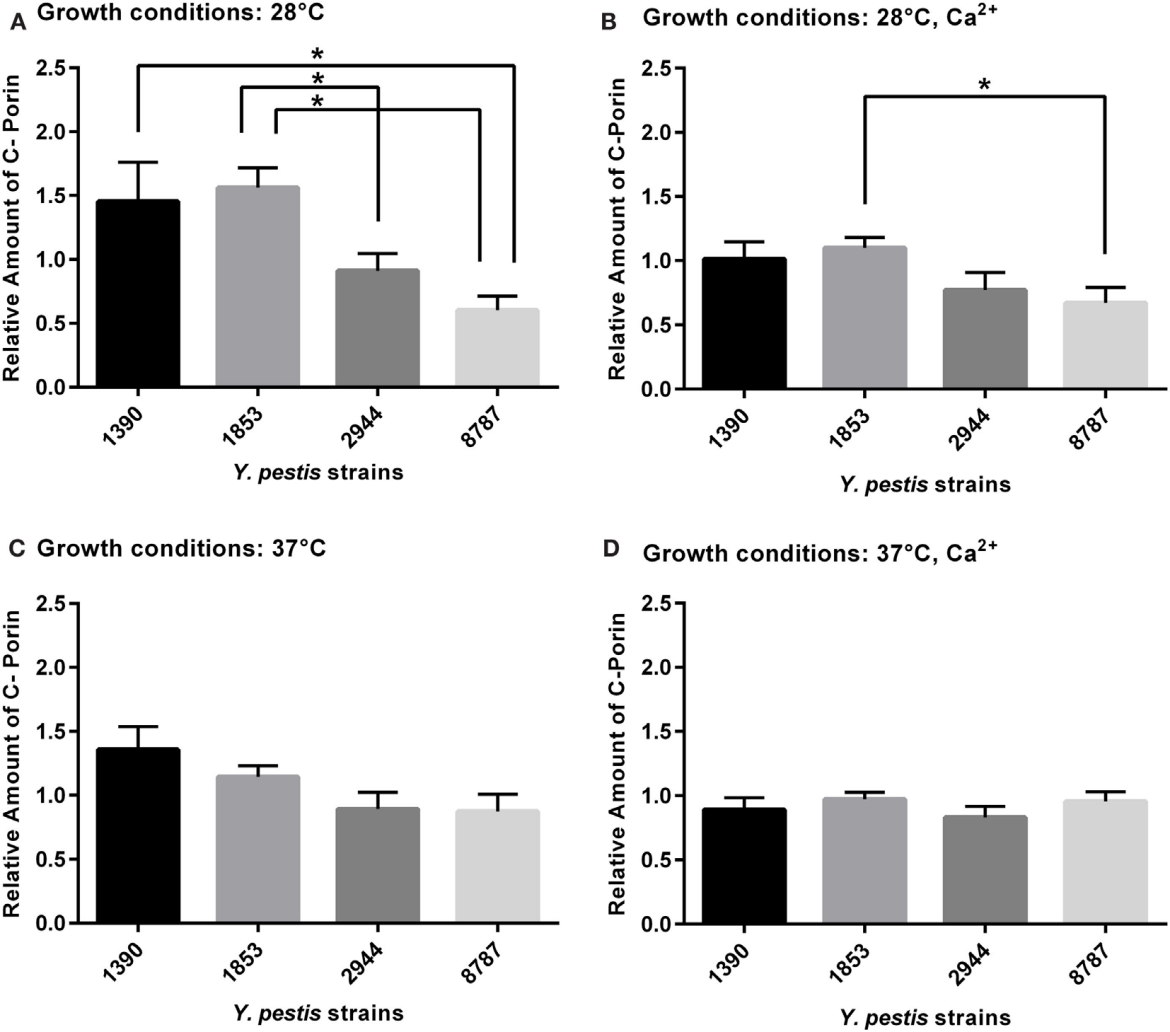
**Relative amount of outer membrane protein C, porin expressed by *Y. pestis* strains 1390, 1853, 2944, and 8787 at physiological growth conditions (A) 28°C; (B) 28°C, Ca^2+^; (C) 37°C; and (D) 37°C, Ca^2+^**. The relative amount of protein expressed was calculated from immunostained bands, by dividing the optical density of the band by the optical density of the corresponding 30 μg total protein standard. Data represent the mean + SEM. *Indicates significant differences (*p* < 0.05).

Two-way ANOVA revealed significant differences in expression of outer membrane protein C, porin, between strains (*F*_3, 63_ = 8.53, *p* = 0.0001). The largest significant differences were observed at 28°C (Figure 4A). The mean amount of outer membrane protein C, porin was significantly higher in strain 1853 than strains 2944 (*p* = 0.02) and 8787 (*p* = 0.002), and in strain 1390 than strain 8787 (*p* = 0.041). The amount of outer membrane protein C, porin was significantly higher in strain 1853 than 8787 at 28°C with Ca^2+^ (*p* = 0.025, Figure 4B). Statistically significant differences were not observed between strains grown under other conditions (Figures 4C,D).

### Tellurium-Resistance Protein

**Figure 5:**
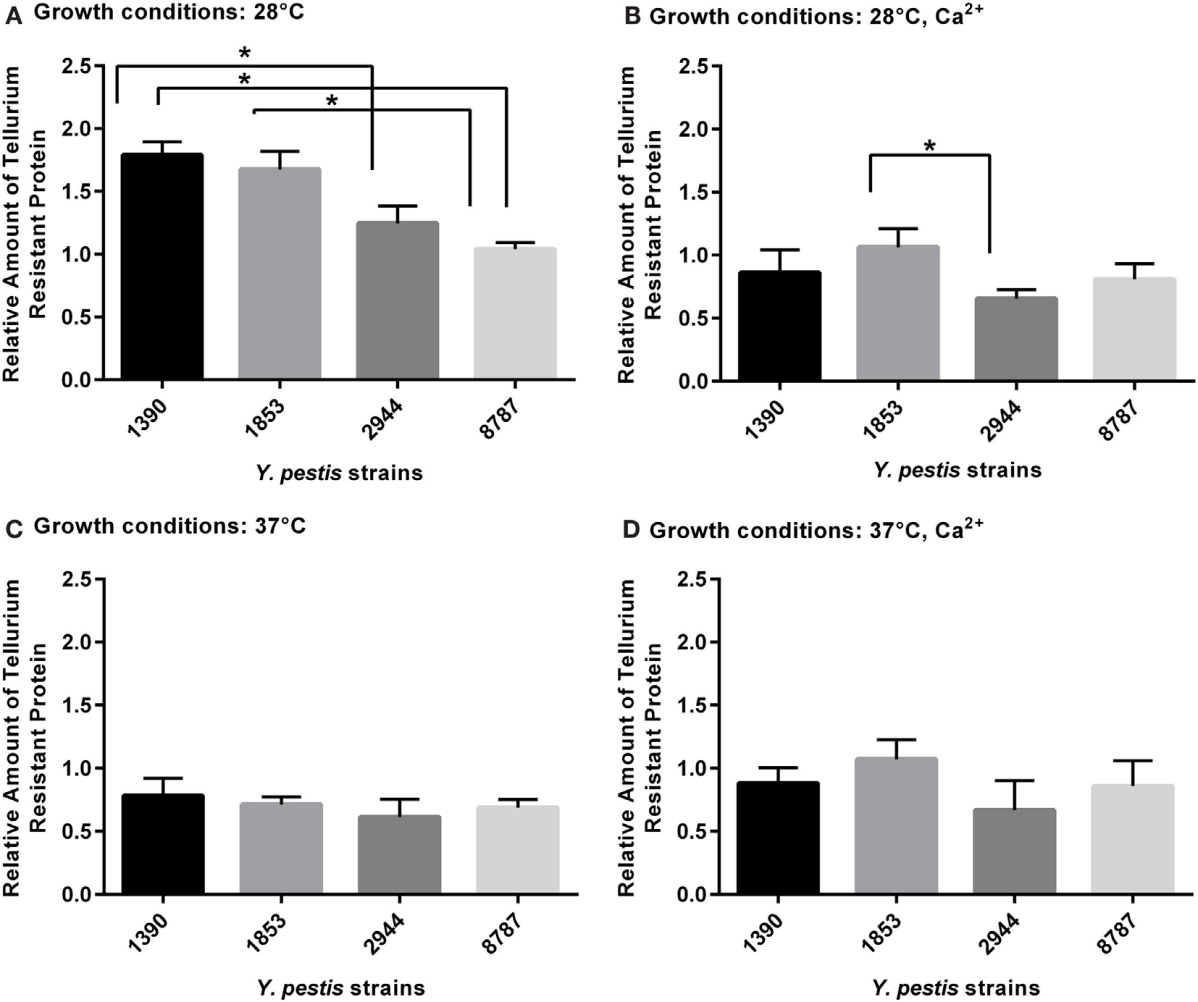
**Relative amount of tellurium-resistance protein expressed by *Y. pestis* strains 1390, 1853, 2944, and 8787 at physiological growth conditions (A) 28°C; (B) 28°C, Ca^2+^; (C) 37°C; and (D) 37°C, Ca^2+^**. The relative amount of protein expressed was calculated from immunostained bands, by dividing the optical density of the band by the optical density of the corresponding 30 μg total protein standard. Data represent the mean + SEM. *Indicates significant differences (*p* < 0.05).

For the tellurium-resistance protein, the effects of strain and growth conditions on expression (respectively *F*_3, 63_ = 5.49, *p* = 0.002; *F*_3, 63_ = 21.03, *p* = 0.0001) were significant by two-way ANOVA. The mean amount of protein at 28°C was significantly higher in strain 1390 than strains 2944 (*p* = 0.019) and 8787 (*p* = 0.001), and the amount in strain 1390 was significantly higher than in strain 8787 (*p* = 0.006). At 28°C with Ca^2+^, the mean value of protein in 1853 was significantly higher than 2944 (*p* = 0.044) (Figures 5A–D); no other significant differences were observed.

### DNA-Binding Protein H-NS

**Figure 6:**
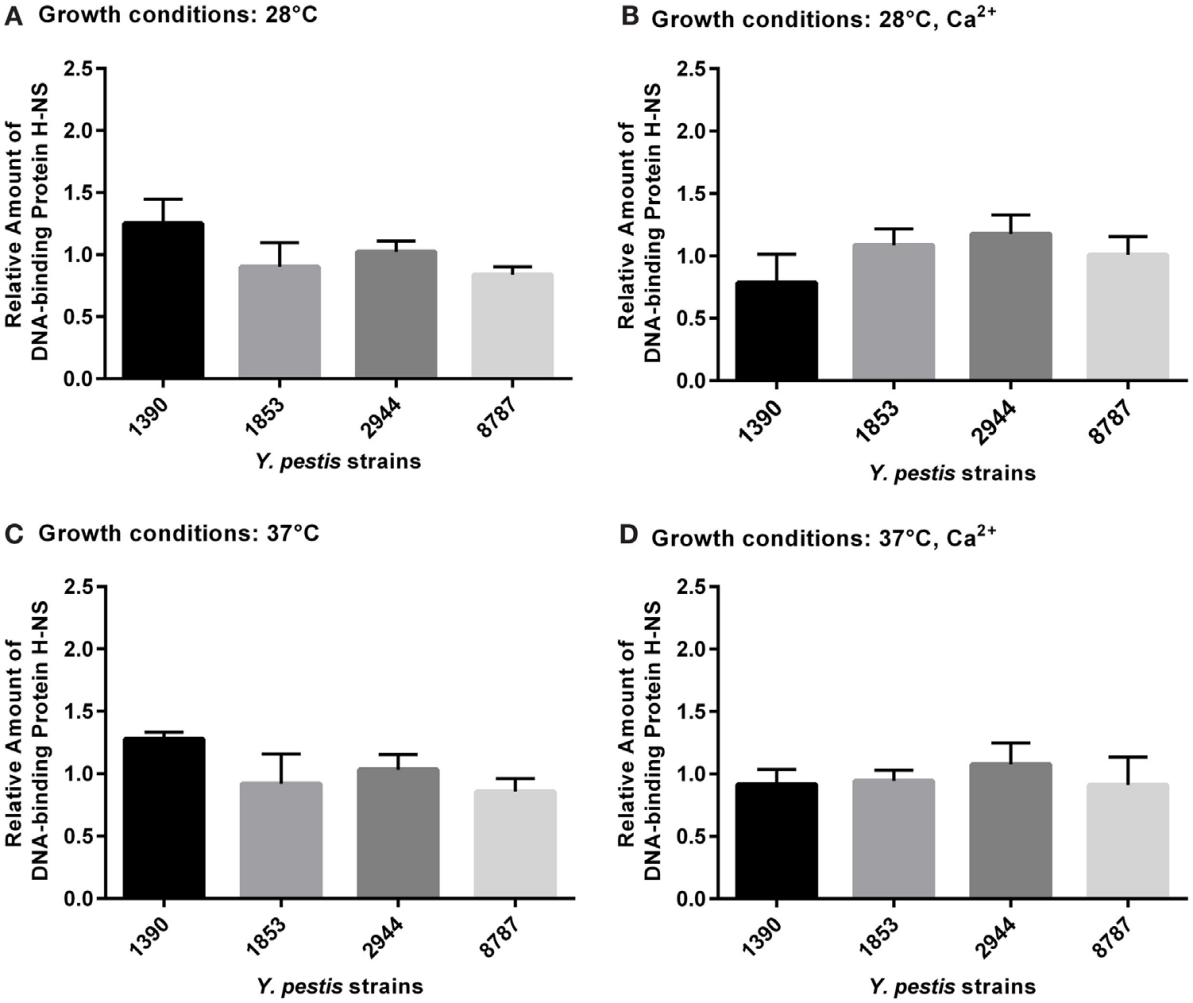
**Relative amount of DNA-binding protein H-NS expressed by *Y. pestis* strains 1390, 1853, 2944, and 8787 at physiological growth conditions (A) 28°C; (B) 28°C, Ca^2+^; (C) 37°C; and (D) 37°C, Ca^2+^**. The relative amount of protein expressed was calculated from immunostained bands by dividing the optical density of the band by the optical density of the corresponding 30 μg total protein standard. Data represent the mean + SEM. *Indicates significant differences (*p* < 0.05).

None of the differences in expression of DNA-binding protein H-NS between strains or under different growth conditions were significant by ANOVA (Figures 6A–D).

### *In vitro* Cytotoxicity Assay

**Figure 7:**
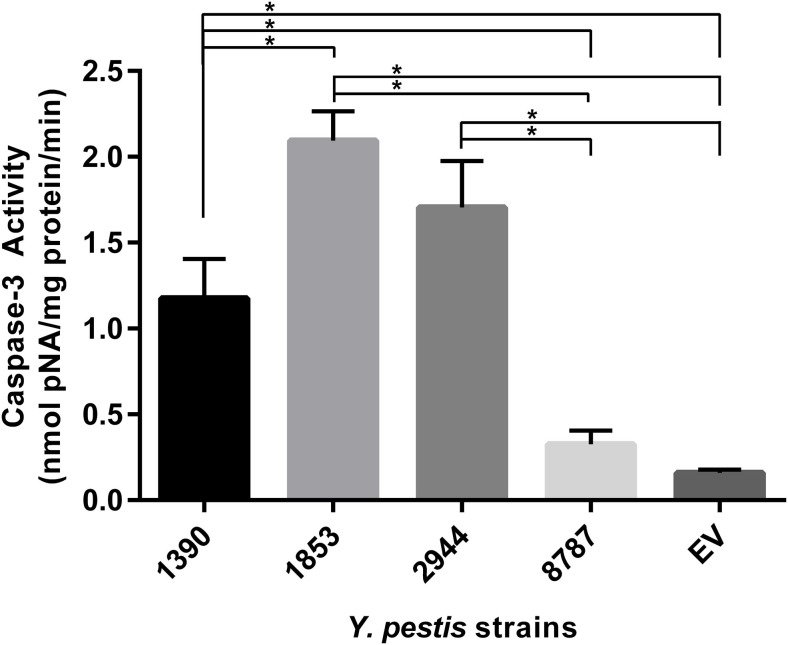
**Macrophage caspase-3 activity under the influence of *Y. pestis* strains 1390, 1853, 2944, 8787, and EV**. *In vitro* cytotoxicity of *Y. pestis* strains and the vaccine strain EV was evaluated based on their ability to induce apoptosis in macrophage cultures. Data represent the mean + SEM. *Indicates significant differences (*p* < 0.05).

Caspase-3 activity of *Y. pestis* strains was compared using one-way ANOVA; activity was significantly different between strains (*F*_4_, _19_ = 22.15, *p* = 0.0001; Figure 7). The highest activity of caspase-3 was observed in strain 1853, which significantly exceeded the activity in strains 1390 (*t* = 3.24, *p* = 0.018), 8787 (*t* = 9.35, *p* = 0.0001), and EV (*t* = 11.20, *p* < 0.0001). Caspase-3 activity in strain 2944 was significantly higher than in strains 8787 and EV (*t* = 5.69, *p* = 0.001). Caspase-3 activity was also significantly higher in strain 1390 than strains 8787 (*t* = 3.55, *p* = 0.012) and EV (*t* = 4.48, *p* = 0.004) (Figure 7). No other significant differences were observed (by two-tailed *t*-test).

## Discussion

Genetic polymorphisms are often associated with various *Y. pestis* strains isolated from natural foci in the Republic of Georgia, but it is not known if these polymorphisms affect gene expression or if there are differences between the proteomes of various strains (42, 43). The results of this study demonstrate that strains of *Y. pestis* isolated from natural foci in the Republic of Georgia differ at the proteomic level as well as the genetic level.

The main goal of this research was to identify a set of candidate proteins that are differentially expressed across the different Georgian *Y. pestis* isolates. A combination of 2-D electrophoresis and MS was used to identify several candidate proteins. However, 2-D electrophoresis with silver staining is not a strong quantitative approach, even when analyzed with even the most sophisticated software; therefore, further validation was required. To this end, we used quantitative Western blotting to measure the levels of proteins (the final products of gene expression). From candidate proteins, the following proteins were chosen as the focus of the experiments: F1 antigen; tellurium-resistance protein; outer membrane protein C, porin, and DNA-binding protein H-NS.

F1 antigen is widely accepted as a virulence factor of Y. pestis. F1 is encoded by the caf1 gene located on the large 100-kb pFra plasmid, which is unique to *Y. pestis*. F1 antigen is synthesized as a 15–16 kDa monomer and forms a large homopolymer (>200 kDa) on the bacterial cell surface in a stacked ring structure composed of heptamers (44, 45). The secretion and assembly of F1 require the *caf1M* and *caf1A* genes, which are homologous to the chaperone and usher protein families required for biogenesis of pili. F1 may be involved in the ability of *Y. pestis* to prevent uptake by macrophages, thus protecting *Y. pestis* from the host’s innate immune response (45). F1-antigen is also known to inhibit bacterial adhesion to epithelial cells (45).

As a differentially expressed protein, F1 antigen was detected on gels with bacterial extracts grown at 37°C and at 37°C with Ca^2+^. Western blotting analysis verified that the mean amount of F1 antigen was significantly higher in strain 1390 than in strain 2944 and 8787 at 37°C. The mean amounts of F1 antigen expressed at 37°C with Ca^2+^ were not significantly higher in strains 1390 and 1853 than strains 2944 and 8787. These results indicate that expression of F1 antigen differs from strain to strain. Further, the F1 antigen has been well-documented to be temperature regulated. Prior work demonstrates enhanced F1 expression at >35°C. Considering that our findings revealed enhanced expression at 37°C relative to 28°C (most evident when grown in the presence of calcium), this provides additional authenticity to our results (46).

Tellurium is a trace element that belongs to the same chemical group as selenium, sulfur, and oxygen. Tellurite oxyanions are highly toxic for most forms of life even at micromolar levels (47). Toxicity of tellurium may be mediated partly by reactive oxygen species that are generated as by-products of tellurite reduction (48). The primary functions of tellurium-resistance proteins are not known, although some sources suggest that they are involved in detoxification of antimicrobial compounds produced by host macrophages (47). *Y. pestis* cells in natural rodent hosts multiply initially in macrophage phagolysosomes. Survival and multiplication of *Y. pestis* in this new environment likely requires compensatory mechanisms involving expression of specific proteins compared to those expressed during extracellular growth. Indeed, 2-D electrophoresis and MS analysis has shown that intracellular and extracellular *Y. pestis* proteomes differ from each other in the expression of 12 proteins (49). Differentially expressed proteins that were upregulated inside the macrophages include tellurium-resistance proteins and also DNA-binding protein H-NS (49). Since the survival and multiplication of *Y. pestis* inside macrophages may be enhanced tellurium-resistance proteins, the strain-specific variations in tellurium-resistance protein expression might influence virulence during mammalian infection. The results of these experiments suggest that strains 1390 and 1853 are expressing tellurium-resistance protein at higher levels than strains 2944 and 8787.

Iron is an essential element for the survival of many microorganisms and its acquisition by bacteria can be central to the outcome of an infection (45). Recent data indicate that outer membrane proteins, including protein C, are transferrin binding proteins and could be involved in the acquisition of iron for growth within their host (47). Differences in the expression of outer membrane protein C, porin, between the strains were generally analogous to differences in expression of tellurium-resistance protein. Significant differences between the strains were observed at 28°C; the mean amount of protein was higher in strains 1390 and 1853 than strains 2944 and 8787.

DNA-binding protein H-NS belongs to a group of nucleoid-associated proteins, which are associated with the chromosome. They possess substantial non-specific DNA-binding affinity and have two major functions: gene regulation and chromosome organization (50). As mentioned above, this protein in is upregulated in *Y. pestis* inside of macrophages (49). DNA-binding protein H-NS was identified as a significantly differentially expressed protein when analyzed on silver-stained 2-D gels, but further quantitative analysis with Western blotting indicated that differences in expression between strains were not significant. The greatest difference, which was observed between strains 1390 and 8787, was only significant in a one-tailed *t*-test.

It is well documented that cell death plays a central role in host-pathogen interactions by eliminating the pathogen’s replicative niche and/or by eliminating immune cells and evading antimicrobial effector mechanisms (51). In these experiments, the ability to induce apoptosis in macrophage cell cultures was used to determine the toxicity of *Y. pestis* isolates. Macrophages are responsible for detecting, engulfing, and destroying pathogens and their apoptosis terminates the host’s immune response. Caspase-3 is a mediator of the pathogenic effect of *Y. pestis* in the livers of C57BL/6 mice (52); therefore the apoptosis activity, as indicated by caspase-3 expression, could be a measure of the virulence of *Y. pestis* isolates. Caspase-3 activity differed significantly among the study strains: the lowest significant expression was observed in strains EV and 8787, and the highest was observed in 1853. The apoptosis-inducing activities in strains 2944 and 1390 were also significantly higher than in strains 8787 and EV.

The overall comparative analysis of studied strains indicates that strain 8787 is distinct from other strains due to a low expression of virulence factors and cytotoxic activities. According to the neighbor-joining tree generated for 46 *Y. pestis* strains from the NCDCPH, strain 8787 is different from strains 1390, 1853, and 2944. Strains 1853 and 1390 are in close proximity to each other. The levels of expression of F1, tellurium-resistance protein, and outer membrane protein C, porin suggest that strains 1853 and 1390 are close to each other. According to the cytotoxicity assay, strains 1853 and 1390 are significantly different from each other and are both different from 8787 and 2944. Strain 2944 is characterized by a lower expression of the studied proteins and high cytotoxic activity, which is likely driven by factors other than those studied. Thus, the results obtained confirm both relatedness and differences between the strains.

Plasminogen activator, Pla is encoded by the pPCP plasmid and is one of the major virulence factors in *Y. pestis* (53). According to one study, the pPCP plasmid is present in all strains of *Y. pestis* from the NCDCPH collection (25), but in another series of experiments, the plasmid was found only in three strains (2944, 2614, and 790) (25, 30). If the plasmid is only found in three strains, the high cytotoxic activity of strain 2944 could be explained by the presence of the pPCP plasmid. According to the blotting data, the Pla is expressed in all of these strains (data not shown). We also consider the possibility that Pla gene from pPCP plasmid was integrated in to the bacterial chromosome, which has been reported in previous studies.

The proteome of *Y. pestis* as a function of changes in temperature and calcium provides information regarding the expression levels of virulence-associated factors, putative virulence factors, and metabolic and housekeeping proteins, as well as potential novel virulence determinants. Future studies using altered gel formulations to examine lower-molecular-weight proteins, and experiments to control the proteolytic activity of Pla will provide more information about these *Y. pestis* strains. Although, we can infer that there are differences between strains and the functions of the identified differentially expressed proteins, future studies using mutant strains of *Y. pestis* are needed. The proteomic analysis of these strains reported here allows for future comparisons of clinical and environmental isolates across the Caucasus.

## Conflict of Interest Statement

The authors declare that the research was conducted in the absence of any commercial or financial relationships that could be construed as a potential conflict of interest.
